# Phenolic Compounds in Field Horsetail (*Equisetum arvense* L.) as Natural Antioxidants

**DOI:** 10.3390/molecules13071455

**Published:** 2008-07-17

**Authors:** Neda Mimica-Dukic, Natasa Simin, Jelena Cvejic, Emilija Jovin, Dejan Orcic, Biljana Bozin

**Affiliations:** 1 Department of Chemistry, Faculty of Sciences, Trg D. Obradovica 3, University of Novi Sad, 21000 Novi Sad, Serbia. E-mails: simin@uns.ns.ac.yu, ciao.ema@gmail.com; 2 Department of Pharmacy, Faculty of Medicine, Hajduk Veljkova 3, University of Novi Sad, 21000 Novi Sad, Serbia. E-mails: cvejich@hotmail.com, bbozin2003@gmail.com

**Keywords:** *Equisetum arvense* L*.*, NO, DPPH, LP, reducing power

## Abstract

In this paper, the study of antioxidant activity and phenolic composition of three different extracts (EtOAc, n-BuOH and H_2_O) of field horsetail (*Equisetum arvense* L.) is presented. The antioxidant activity has been evaluated measuring the total reducing power (expressed by Ascorbate Equivalent Antioxidant Capacity - AEAC), inhibition of lipid peroxidation, and free radical scavenging capacity (RSC) towards 2,2-diphenyl-1-picrylhydrazyl (DPPH radical) and nitric oxide (NO), respectively. In addition, the total flavonoid content (TFC) and phenolic constituents of each extract have been determined. The results obtained show that the highest RSC regarding both DPPH and NO radicals is expressed by EtOAc extract (EC_50_=2.37 μg/mL and EC_50_=90.07 μg/mL, respectively), and the lowest by H_2_O extract (EC_50_=37.2 μg/mL and EC_50_>333.33 μg/mL, respectively). n-BuOH extract showed the highest total reducing power (AEAC=13.40 μg/mL). Differences in the phenolic composition of examined extracts are found comparing the HPLC chemical profiles. Although, isoquercitrin is the main flavonoid in both EtOAc and n-BuOH extracts, a considerable amount of di-*E-*caffeoyl-meso-tartaric acid was presented in the n-BuOH extract. In H_2_O extract high content of phenolic acids and low percentage of flavonoids were detected.

## Introduction

This study is a continuation of our efforts to evaluate plant extracts as sources of natural antioxidants [1]. *Equisetum* *arvense* L. (Equisetaceae, subgenus *Equisetum*) is a well-known and widespread pteridophyte distributed throughout the Northern Hemisphere. Field horsetail contains more than 10% inorganic substances (two-thirds of which are silicic acid and potassium salts). Also, the drug is rich in sterols: β-sitosterol, campasterol, isofucosterol [2], ascorbic acid, phenolic acids (cinnamic acids, caffeic acid, di-*E*-caffeoyl-*meso*-tartaric acid and 5-*O*-caffeoylshikimic acids), polienic acids, rare dicarboxylic acids (equisetolic acid), flavonoids [3] and styrylpyrones [4]. Flavonoid composition reveals the existence of two chemotypes of *E. arvense* L., one occuring in Asia and North America and the other in Europe. Material from Asia and North America contains luteolin 5-*O*-glucoside and its malonyl ester, whereas European material is lacking these compounds [5]. Di-*E*-caffeoyl-*meso*-tartaric acid is a marker for both chemotypes. The dominant compounds in European plants are quercetin 3-*O*-glucoside, apigenin 5-*O*-glucoside and dicaffeoyl-*meso*-tartaric acid [6]. Sterile stems of *E. arvense* are used in herbal medicine in various countries, constituting the “Equiseti herba” of European Pharmacopeias (DAB 10, Ph.Helv. VII, ÖAB 90, Ph. Pol. III, Ph. Ross 9 and Ph. Hung.). Therapeutic use of *Equisetum* preparations are related to the reputed aquaretic and antihaemorragic properties of the plant. The most recent experimental data shows that ethanol and aqueous extracts of *E. arvense* fertile stems are possessing very high radical scavenging activity towards superoxide anion and hydroxyl radicals [7], but data regarding to antioxidant properties of sterile stems of *E. arvense* are still being scarce. Concerning the complex composition of the plant extracts, their antioxidant activity must be evaluated combining different *in vitro* assays to get relevant data.

With respect to this, the antioxidant activity of three separated fractions of methanolic extract (EtOAc, n-BuOH and H_2_O) of *Equisetum arvense* sterile stems has been investigated in the present study. The antioxidant activity was evaluated measuring the total reducing power, inhibition of induced lipid peroxidation in liposome and free radical scavenging capacity (RSC) regarding DPPH and NO radicals. In addition, the total flavonoid content (TFC) and HPLC chemical profile of phenolic constituents in each extract were determined.

## Results and Discussion

### Chemical composition

**Figure 1:**
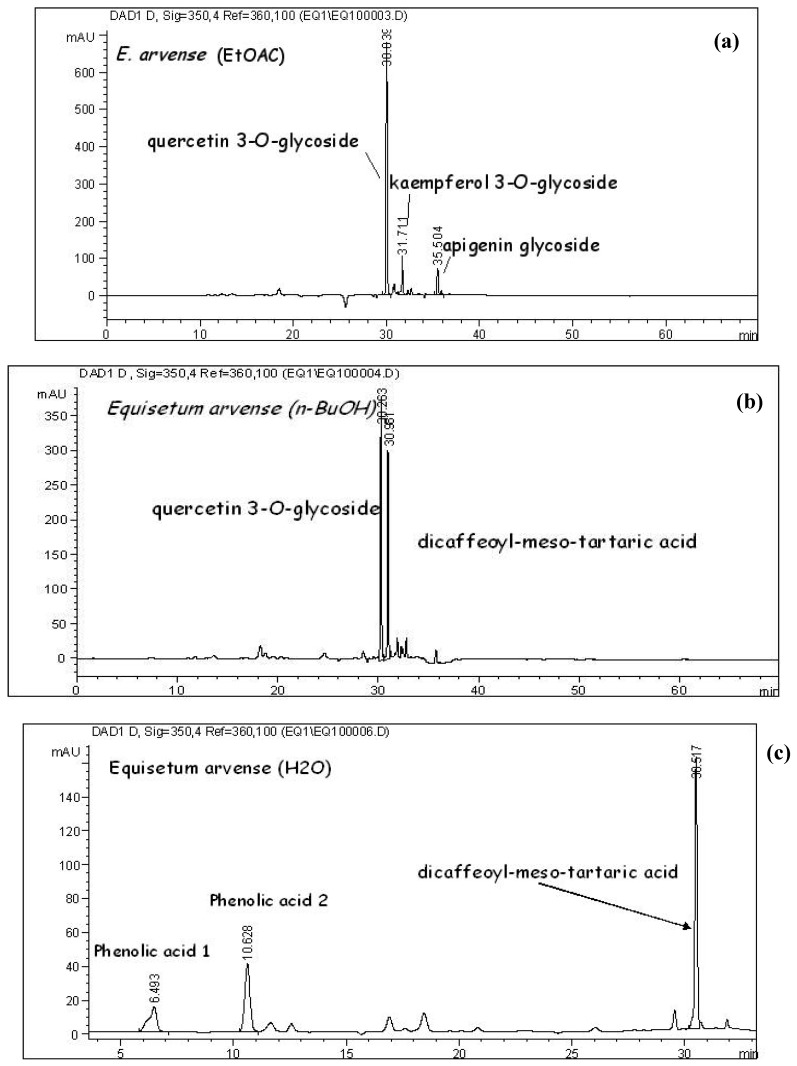
(a) HPLC chromatogram of EtOAc extract of *E. arvense* L. Detection at 350 nm. (b) HPLC chromatogram of n-BuOH extract of *E. arvense* L. Detection at 350 nm. (c) HPLC chromatogram of H_2_O extract of *E. arvense* L. Detection at 350 nm.

Flavonoid glycosides (quercetin 3-O-glucoside, apigenin 5-*O*-glucoside, kaempferol 3-*O*-glycoside) and di-*E-*caffeoyl-*meso*-tartaric acid have been identified by HPLC-DAD analysis of extracts of *E. arvense* native to Vojvodina. These compounds were previously described as the main constituents in European *E. arvense* [5, 6]. The main compound in the EtOAc extract was quercetin 3-*O*-glucoside (isoquercitrin) (152 mg/g DE), while apigenin 5-*O*-glucoside and kaempferol 3-*O*-glycoside were detected in considerable amounts (22.4 mg/g and 26.2 mg/g DE, respectively) (Figure 1a, Table 1). In the *n*-BuOH extract, beside isoquercitrin (382 mg/g DE), di-*E-*caffeoyl-*meso*-tartaric acid was found in high amounts (100 mg/g DE, respectively) (Figure 1b, Table 1). In the H_2_O extract beside di-*E-*caffeoyl-*meso*-tartaric acid (10 mg/g DE), two more phenolic acids (3 and 6 mg/g DE) were detected (Figure 1c, Table 1). While flavonoids were recorded as the main compounds in the EtOAc and *n*-BuOH extracts, phenolic acids were the major constituents in the aqueous extract. The difference between the EtOAc and *n*-BuOH extracts was mainly reflected in the presence of di-*E-*caffeoyl-*meso*-tartaric acid in *n*-BuOH extract and apigenin 5-*O*-glucoside and kaempferol 3-*O*-glycoside in the EtOAc one (Table 1). The highest total flavonoid content was found in the *n*-BuOH extract (135.01mg/g DE) and lowest in the H_2_O extract (4.89 mg/g DE) (Table 2).

**Table 1:** The results of HPLC-DAD phenolic identification and quantification of *E. arvense* extracts

Dominant phenolic compounds	EtOAc(mg/g DE)	*n*-BuOH(mg/g DE)	H_2_O(mg/g DE)
isoquercitrin	152.0	382.0	
apigenin 5-*O*-glucoside	22.40		
kaempferol 3-*O*-glycoside	26.20		
di-*E-*caffeoyl-*meso*-tartaric acid		100.0	10.00
phenolic acid 1			3.00
phenolic acid 2			6.00

### Antioxidant activity

The results of DPPH-RSC, NO-RSC, LP inhibition and reducing power assay are shown in Table 2. The highest DPPH-RSC was expressed by the EtOAc extract (EC_50_=2.37 µg/mL) and the lowest one by the H_2_O extract (EC_50_=37.20 µg/mL). Among synthetic antioxidants the most powerful DPPH scavenger was PG (EC_50_=0.49 µg/mL) whereas BHT expressed the lowest DPPH-RSC (EC_50_=8.26 µg/mL).

The highest NO-RSC (EC_50_=90.07 µg/mL) was found for the EtOAc extract, while the H_2_O extract did not reach 50% of NO neutralization (EC_50_>333.33 µg/mL). Among synthetic antioxidants, PG exhibited the strongest NO scavenging effect (EC_50_=12.08 µg/mL), since BHT and BHA did not reach EC_50_ value.

All extracts scavenged both DPPH and NO radicals dose-dependently.

The highest reduction of Fe(II)/ascorbate induced LP in liposome was obtained by the EtOAc extract (EC_50_=14.50 µg/mL) and the lowest one by the H_2_O extract (192.31 µg/mL). The LP inhibitions exhibited by EtOAc extract and by BHA were almost the same (EC_50 _(BHA)=15.06 µg/mL) (Table 1).

With regards to reducing power assay, the *n*-BuOH extract possesses an approximately eight times higher AEAC (13.47 µg/mL) than the other two.

Comparing the chemical composition with the particular antioxidant assays the following conclusions could be made: the mixture of isoquercitrin and two additional flavonoids is obviously most responsible for the highest scavenging and LP-inhibitory effect, shown by the EtOAc extract.

This is also supported by the very low antioxidant capacity of the aqueous extract, in which flavonoids have been detected only in traces. On the other hand, the highest AEAC in the *n*-BuOH extract could be attributed to the huge amount of total flavonoids and the very high ratio of isoquercitrin and di-*E-* caffeoyl-*meso*-tartaric acid. It can be presumed that later compounds can act as powerful Fe(III) reducing agents. A strong relationship between total phenolic content and AEAC was reported earlier [8].

**Table 2:** Total flavonoid content, reducing power and antioxidant activity (DPPH-RSC, NO-RSC, LP) of examined *E. arvense* extracts and synthetic antioxidants (BHA, BHT and PG).

Sample	TFC(mg/g DE)	RP[AEAC (µg/mL)]	DPPH-RSC [EC_50 _(µg/mL)]	NO-RSC[EC_50 _(µg/mL)]	LP[EC_50 _(µg/mL)]
EtOAc extract	17,47	1,57	2.37	90.07	14.50
*n*-BuOH extract	135,01	13,47	7.16	105.90	150.47
H_2_O extract	4,89	1,56	37.20	>333.33	>192.31
BHA	-	-	2.02	I *_max_*=38.01% (C*_final_*=42.04 µg/mL)	15.06
BHT	-	-	8.26	I_max_=24.47% (C_final_=73.47 µg/mL)	93.45
PG	-	-	0.49	12.08	37.92

TFC is expressed by mg rutin/g dry extract, RP is expressed by AEAC (µg/mL), DPPH-RSC, NO-RSC and LP are expressed by EC_50_ (µg/mL)

Among all tested extracts and synthetic substances PG was the strongest antioxidant. However, the antioxidant activity of EtOAc extract was higher or close to that of commercial antioxidants BHA and BHT. Moreover, EtOAc and n-BuOH extracts demonstrated strong scavenging of NO, compared with synthetic antioxidants. This is very important as it is known that beside its significant physiological role, nitric oxide is highly toxic in excessive amounts, especially after oxidation into peroxynitrite [9]. Thus, it is tempting to conclude that beside the general antioxidant activity, the NO scavenging property of *E. arvense* is very important in the protection against many pathological conditions.

## Conclusions

In conclusion, the results of the antioxidant effect of *E.arvense* extracts, obtained with four different methods of assessment, point out strong protective activity against free radicals, lipid peroxidation and oxidative agents. Together with previously reported data, the obtained results provide and advance knowledge of antioxidant properties of *E.arvense*.

## Experimental

*Plant material:* The sterile stems of wild growing *E. arvense* were collected in May of 2002 from the Fruska Gora mountain in the Vojvodina province in Serbia. Aerial parts of the plants were used for the experiment. Voucher specimens (*E. arvense* L. N° 2-1965, Fruska Gora-Vrdnik, UTM 34TDQ09, det: Goran Anackov) were confirmed and deposited at the Herbarium of the Department of Biology and Ecology (BUNS Herbarium), Faculty of Natural Sciences, University of Novi Sad.

*Chemicals:* Petroleum ether, methanol, ethyl-acetate, n-buthanol, acetic acid, trichloroacetic acid (TCA), H_3_PO_4, _NaH_2_PO4, Na_2_HPO4 and ascorbic acid were purchased from Lach-Ner s.r.o. (Neratovice, Czech Republic). AlCl_3_x6H_2_O, CH_3_COONa, FeSO4, HClO_4_, sodium nitroprusside, K_3_[Fe(CN)_6_] and FeCl_3_ were provided by Reanal (Budapest, Hungary). Phosphoric acid (HPLC purity), methanol (HPLC purity), EDTA, 2-thiobarbituric acid (TBA) and 2,2-diphenyl-1-picrylhydrazyl (DPPH) were obtained from Sigma (St. Louis, MO, USA). 3,5-di-*tert*-butyl-4-hydroxytoluene (BHT) and propylgallate (PG) were purchased from Aldrich (Taufkirchen, Germany) and 3-*tert*-butyl-4-hydroxyanisole (BHA) and sulfanilamide from Fluka Chemie GmbH (Buchs, Switzerland). Preparation of liposomes “PRO-LIPO S” pH 5-7 was obtained from Lucas- Meyer (Hamburg, Germany) and *N*-(1-naphtyl)-ethylenediamine dihydrochloride (NEDA) from Alfa Aesar (Karlsruhe, Germany). Rutin trihydrate was provided by Carl Roth GmbH, Karlsruhe, Germany).

*Preparation of extracts and determination of total flavonoid content:* 100 g of air-dried and powdered plant material was macerated with petroleum ether overnight and afterwards with 70% MeOH (24 h). After filtration, the methanolic extract was concentrated to dryness using a rotary evaporator. The dry residue was dissolved in hot water and then separated by liquid-liquid extraction into CHCl3, EtOAc and *n*-BuOH extracts. These extracts were evaporated to dryness and the dry residues were re-dissolved in 70% MeOH to obtain mass concentration: 0.5, 1.0, 5.0 and 10%. The obtained extracts were used in further experiments.

*Total flavonoid content:* Determination of total flavonoid content (TFC) in the examined extracts was based on flavonoid affinity to form a complex with AlCl_3_ [10]. 100µL of each extract (10%) was made up to a final volume of 10 mL with reaction medium (MeOH/H_2_O/CH_3_COOH=14:5:1). From this solution, 1 mL was taken and diluted with distilled water to 20 mL. 

Prepared solution (10 mL) was mixed with AlCl_3 _reagent (5 mL, 133 mg of AlCl_3_x6H_2_O and 400 mg of CH_3_COONa dissolved in 100 mL H_2_O). After 5 min, the absorbance of the resulting solutions was measured in comparison with the blank (containing the same chemicals, except for the sample) at 430 nm (CECIL 2021 spectrophotometer). TFC was calculated on the basis of the calibration curve of rutin and expressed by mg rutin/g dry extract (mg/g DE). For each sample three replicates were carried out.

*HPLC analysis:* The sample was filtrated through 0.45 µm cellulose membrane filter (Econofilters, Agilent Technologies, Germany) and an aliquot (5 µL) of the sample was injected in the HPLC. The reversed-phase HPLC system (Agilent 1100 Series, Germany) consisting of an Agilent Technologies HPLC system (Agilent 1100 Series, Germany), was equipped with a binary pump, UV-diode array detector, autosampler and ChemStation for LC 3D (Rev. A.09.03.) software. The chromatographic conditions were: temperature 15°C thermostated, column Zorbax (SB-C18 4.6 x 150 mm, 5-µm), guard column Zorbax (SB-C18 4.6 x 12.5 mm, 5-µm). Two solvents were used for the gradient elution: (A) 0.15 % phosphoric acid in H_2_O: MeOH (77:23) (pH = 2) and (B) methanol. The elution profile was: isocratic: 0 - 3.6 min 100A; gradient: 3.6 min. 100 % A - linear - 24.0 min. 80.5% A - isocratic - 30 min - linear - 60 min. 51.8 % A - linear - 67.2 min. 100 % B. The flow rate was 1.0 mL/min.

Detection and quantification were carried out using 330 and 350 nm as preferred wavelengths. Peak purity and identity were checked by comparison of the UV spectra (DAD-detector) with those of the reference substances and with literature data [6, 11]. Quantification of dominant compounds (mg/g DE) was performed by HPLC method using a single point calibration with an external standard. Quercetin 3-O-rutinoside (rutin) was used as standard compound for quantification of flavonols, apigenine for quantification of flavons and chlorogenic acid for phenolic acids.

*Scavenging of the stable radical 1,1-diphenyl-2-picrylhydrasyl – DPPH˙ assay:* DPPH˙-assay was performed as described before [12], with small modifications [1b]. 10 μL of each extract (1.25 - 62.5 µg/mL) was mixed with 90 µmol/L DPPH˙ in methanol (1.0 mL) and made up with pure methanol to a final volume of 4.0 mL. The mixtures were shaken vigorously and were stored in dark for 60 min at room temperature. This is done to reach the steady state of the reaction. After incubation the absorbance was measured at 515 nm (CECIL CE2021 spectrophotometer). All reactions were carried out in triplicate. Commercial synthetic antioxidant: BHT, BHA and PG were used as positive controls. The DPPH˙ scavenging activity was expressed by radical scavenging capacity using the following equation:

DPPH − RSC(%) = 100 * (A_0_ − A_1_/A_0_)

where *A*_0_ was the absorbance of the control reaction (full reaction, without the tested extract or compound) and *A*_1_ was the absorbance in the presence of the scavenger.

The EC_50_ values (concentration of extract in the reaction mixture needed to decrease by 50% the initial DPPH˙ concentration) were determined by polynomial regression analysis of the obtained DPPH-RSC values (software ORIGIN 2001).

*Determination of LP inhibition in liposome:* The extent of Fe(II)/ascorbate induced LP was determined by TBA-assay [13]. The commercial liposome preparation “PRO-LIPO S” pH 5-7 was used as a model system of biological membranes. The liposomes, 225-250 nm in diameter, were obtained by dissolving the commercial preparation in distilled water (1:10) in an ultrasonic bath. The reaction mixture contained liposome suspension (60 µL), FeSO4 (20 µL, 0.01 mol/L), ascorbic acid (20 µL, 0.01 mol/L) and examined extract (10 µL, final concentration 9.62 – 192.31 µg/mL) and made up with NaH_2_PO4-Na_2_HPO4 buffer (2.89 mL, 0.067 mol/L, pH 7.4) to start the peroxidation. After 60 min at 37 ºC the reaction was terminated with TBA-reagent (2.0 mL, 10.4 mL 60% HClO4, 3 g TBA and 120 g 20% trichloroacetic acid (TCA) dissolved in 800 mL dH2O) and EDTA (0.2 mL, 0.1 mol/L). Tubes were heated at 100 ºC for 20 min and cooled immediately. The mixtures were centrifuged at 4000 rpm for 10 min. The content of the MDA (TBARS) was determined by measuring the absorbance at 532 nm (CECIL CE2021 spectrophotometer). The results were compared with commercial synthetic antioxidants: BHT, BHA and PG. The percentage of LP inhibition was calculated by the following equation:

I(%) = (A_0_ − A_1_)/ A_0_ *100

where *A*_o_ is the absorbance of the control reaction (full reaction, without the tested extract or compound) and *A*_1_ is the absorbance in the presence of the inhibitor. A higher percentage indicates a higher LP inhibition.

The EC_50_ value, which represented the concentration of the extract that caused 50% of LP inhibition, was determined by polynomial regression analysis of the obtained I values (software ORIGIN 2001).

*NO scavenging activity:* NO-RSC was evaluated by measuring the accumulation of nitrite (formed by the reaction of NO with oxygen), according to the Griess reaction [14]. NO was generated by sodium nitroprusside in buffered aqueous solution. Each prepared extract (10 μL) was mixed with fresh prepared solution of sodium nitroprusside (1.5 mL, 0.01 mol/L in NaH_2_PO4-Na_2_HPO4 buffer, 0.067 mol/L, pH 7.4) and NaH_2_PO4-Na_2_HPO4 buffer (1.5 mL, 0.067 mol/L, pH 7.4). These mixtures were incubated at 25°C for 60 min. Each reaction mixture (1 mL) was mixed with Griess reagent (1 mL, 0.1% *N*-(1-naphtyl)-ethylenediamine dihydrochloride [NEDA] in distilled water and 1% sulfanilamide in 5% H_3_PO_4_*, ana partes*). Reduction of nitrite by the extracts was determinated spectrophotometrically at 546 nm, by measuring decreased of absorbance of the reaction mixtures regarding the control (containing the same chemicals, except for the sample). For each sample three replicates were carried out. RSC was calculated by following equation:

NO − RSC(%) = 100 −(100 * A_1_ / A_0_)

where *A*_o_ is control and *A*_1_ is a sample solution absorbance. The EC_50_ values were determined by polynomial fitting of the inhibition values (RSC) using software ORIGIN 6.1.

*Reducing power assay:* Reducing power (RP) of all extracts was determined according to the method of Yen and Chen [15], with some modifications. RP was expressed in relation to the reducing power of ascorbic acid as a positive control (AEAC - Ascorbate Equivalent Antioxidant Capacity). Each prepared extract (10 μL) was mixed with K_3_[Fe(CN)_6_] (1 mL, 1%) and NaH_2_PO4-Na_2_HPO4 buffer (1 mL, 0.2 mol/L, pH 6.6). These mixtures were incubated at 50°C for 30 min. Afterwards, trichloroacetic acid (1mL, 10%) was added. Then the mixtures were centrifuged at 3000 rpm for 10 min. Finally, the supernatant fractions (1 mL) were mixed with distilled water (1 mL) and FeCl_3_ (0.2 mL, 0.1%). The absorbances of resulting solutions were measured at 700 nm. For each sample three replicates were carried out. Increased absorbances of the reaction mixture indicated increased reduction power. AEAC was calculated by the following equation:

AEAC = C_A_ * A_S_ / A_A_

where C_A_- final concentration of ascorbic acid in µg/mL, A_S_- absorbance of the sample, A_A_- absorbance of ascorbic acid.
